# COVID-19 vaccination attitudes and acceptance among people with serious mental illness

**DOI:** 10.3389/fpsyt.2024.1535780

**Published:** 2025-01-22

**Authors:** William Small, Caroline Silva, Rachel Johnson, Vincent Betti, Antoinette Nguyen, Lauren Todd, David Jacobowitz, J. Steven Lamberti

**Affiliations:** ^1^ Department of Psychiatry, University of Rochester Medical Center, Rochester, NY, United States; ^2^ University of Rochester School of Medicine and Dentistry, Rochester, NY, United States

**Keywords:** Covid-19, vaccination, community health, preventative health, psychiatry, serious mental illness

## Abstract

**Objective:**

This study examines attitudes towards COVID-19 vaccination among a diverse cohort of adults with serious mental illness (SMI), participant characteristics that are associated with vaccine acceptance, and barriers to COVID-19 vaccination among this population.

**Methods:**

A 28-item questionnaire was administered to 185 adults with SMI receiving care at a university-based outpatient psychiatric clinic. Variables included demographics, health behaviors, and vaccination status. Chi-square tests were used for categorical demographic comparisons on binary COVID-19 vaccine status.

**Results:**

Female participants were more likely to have received COVID-19 vaccination (77.6%) than male (55.7%) participants. White (73.3%) and Hispanic/Latino (81.8%) participants were more likely to have received vaccination than Black/African American (54.9%) participants. Participants who reported having seen a primary care provider (PCP) within the past two years were more likely to be vaccinated (72.1%) than those who had not (41.7%). Participants who reported having received an influenza vaccine in the past two years were more likely to be vaccinated (80.2%) than those who had not (41.8%). Participants who had not been vaccinated were more likely to report greater concerns about all potential barriers to vaccination, including concerns about side effects, cost, health effects, and distrust of clinicians and governments.

**Conclusions:**

The overall vaccination rate of study participants with SMI was similar to that of the general population. Efforts to enhance engagement in primary care may help improve preventative health behaviors in people with SMI.

## 1 Introduction

Coronavirus disease 2019 (COVID-19) has resulted in 6.5 million deaths globally (1), over 1 million deaths in the United States (US) (2), and over 5 million hospital admissions (3). The impact of COVID-19 on US population health is not limited to physical health; the US Census Bureau’s Household Pulse Survey showed that an increasing number of adults experienced a positive screening result for depression and anxiety during the COVID-19 pandemic (4). Individuals living with serious mental illness (SMI), defined as a psychiatric diagnosis that results in chronic functional impairment or disability, were especially impacted by the COVID-19 pandemic, with one UK study showing a significant decrease in the number of primary care visits for those with SMI during the pandemic (5) and another study showing that over 40% of adults with SMI experienced a mental health deterioration during the pandemic (6). Additionally, recent research suggests that schizophrenia is a risk factor for mortality in patients with COVID-19 (7, 8).

To combat the public health risk of COVID-19, multiple vaccinations against COVID-19 were developed and approved by the FDA in 2021, including Pfizer-BioNTech, Moderna, Jansen/J&J, and Novavax vaccines. Although these vaccines were distributed free of charge to the general population in 2021 and 2022, there has been a significant degree of hesitancy to accept vaccination (9), and the vaccine has become a politicized issue (10). Initial research examining COVID- 19 vaccine hesitancy among the general US population found that factors associated with vaccine hesitancy included younger age, Black race, and lower education (11). More recent studies show that, while vaccine acceptance has risen over time, race and gender, specifically Black race and female gender, have continued to correlate with increased vaccine hesitancy (9). Little research has examined, however, factors affecting vaccine acceptance among people with SMI.

The purpose of this study is to determine attitudes towards COVID-19 vaccination among a diverse cohort of adults with serious mental illness, participant characteristics associated with vaccine acceptance, and barriers to COVID-19 vaccination among this population. By better understanding factors and attitudes associated with vaccine hesitancy, public health officials can more effectively tailor outreach and education efforts to this particular patient population and address disparities in access and understanding.

## 2 Methods

### 2.1 Procedures

A survey was administered from June 2021 through July 2022 to English and Spanish-speaking adult outpatients receiving hospital-based outpatient mental health services for severe mental illness (SMI). Of note, the US Food and Drug Administration (FDA) granted emergency use authorization on December 10, 2020, to the Pfizer-BioNTech Vaccine, December 17, 2020 to the Moderna vaccine, and February 27, 2021 to the Janssen (Johnson & Johnson) vaccine. All US states had opened vaccine eligibility to residents aged 16 and over by April 19, 2021. Thus, the survey was administered after the COVID-19 vaccine became widely available.

Study procedures were approved by the Institutional Review Board at the University of Rochester. A waiver of documentation of consent was approved because the survey did not collect any personal identifiers nor any information about sensitive or illegal behaviors. Participants were provided an information sheet detailing the nature of the survey, describing data storage methods, and explaining that no protected health information would be collected. Research assistants were available to answer any questions about data and privacy.

Participants were eligible to participate if they were age ≥18, English- or Spanish-speaking, and currently enrolled in outpatient mental health services at a university clinic enrolling patients with SMI. Though diagnostic data were not collected in this survey, common diagnoses treated at this clinic include schizophrenia, schizoaffective disorder, bipolar disorder, and severe major depressive disorder. This clinic enrolls patients with co-morbid substance use disorders and SMI, but not isolated primary substance use disorder. Participants were recruited via IRB-approved flyers, tabling in clinic lobbies, and clinician referrals. Participants completed surveys in their primary language (English or Spanish) independently and returned completed surveys to research assistants in the lobby. Participants received $5 cash compensation for participating in the survey.

All participants in the study were eligible to receive primary care services at the University of Rochester’s Medicine in Psychiatry Service (MIPS) primary care office in a unique model of co-located, integrated psychiatric and medical care. This model allows for ease of access to primary care and the ability to receive all care at one physical site. In addition, this integrated care model enables clinicians in psychiatry and medicine to work together to provide comprehensive services to individuals with SMI.

### 2.2 Participants

Participants were 185 adults (54.1% female; 43.2% male) receiving outpatient services for severe mental illness. This number of participants represents approximately 10% of the clinic’s total patient population. Most participants were aged 46-60 years (39.5%), with 17.8% aged 18-30 years, 23.8% aged 31-45 years, and 17.8% over 60 years of age. The majority of participants identified as White (40.5%), with 28.6% Black/African American, 24.3% Hispanic/Latino, 1.6% American Indian/Alaskan Native, 0.5% Asian, and 1.1% multiracial (3.2% prefer not to answer) (1). The most common highest levels of education were high school graduate (25.4%) or some college (23.8%). Surveys were completed in English (80%) and Spanish (20%). See **Table 1** for demographics.

**Table 1:** Demographics.

		n	%
**Age**	18-30 years	33	17.8
	31-45 years	44	23.8
	46-60 years	73	39.5
	Over 60 years	33	17.8
	Prefer not to answer	2	1.1
**Sex**	Male	80	43.2
	Female	100	54.1
	Other/non-binary	3	1.6
	Prefer not to answer	2	1.1
**Race**	White	75	40.5
	Black/African-American	53	28.6
	Asian	1	.5
	American Indian/Alaska Native	3	1.6
	Hispanic/Latino	45	24.3
	Multiracial	2	1.1
	Prefer not to answer	6	3.2
**Education**	Did not finish high school	37	20.0
	High school graduate	47	25.4
	Some college	44	23.8
	Associate Degree	15	8.1
	Bachelor’s Degree	19	10.3
	Graduate Degree	11	5.9
	Prefer not to answer	12	6.5

### 2.3 Measures

Participants were asked to complete a 28-item pen-and-paper questionnaire exploring health perceptions and behaviors, and their correlations with respondent characteristics. Variables included demographic categories (age range, gender identity, race, ethnicity, education level), engagement in primary care, history of influenza vaccine, and presence of a chronic health condition. Participants also rated, on a 5-point Likert scale, single-items assessing their perceptions of physical health (1-Poor to 5-Excellent), mental health (1-Poor to 5-Excellent), perceptions of COVID-19 risk to their overall health (1-Not at all a risk to 5-Severe risk), worry about getting sick with COVID-19 (1-Not at all worried to 5-Extremely worried), and impact of the pandemic on their life and functioning (1-Not at all to 5-Extremely). In addition, participants assessed various barriers to receiving the COVID-19 vaccine on single-item 4-point Likert scales (1-No barrier to 4-Major barrier), including: side effects, cost, effect on mental health, effect on medications, distrust of federal, state, or local government, distrust of vaccine manufactures, and distrust of clinicians administering the vaccine. Participants indicated whether they had already received at least one dose of the COVID-19 vaccine (yes/no); for those who had not received the COVID-19 vaccine, likelihood of accepting COVID-19 vaccine was rated on a 5-point Likert scale (1-Definitely will not to 5-Definitely will).

### 2.4 Data analytic plan

Chi-square tests were used for categorical demographic comparisons (gender, race/ethnicity, age, education) on binary (yes/no) COVID-19 vaccine status. One-way ANOVA or independent t-tests were used for demographic comparisons on likelihood of getting the COVID-19 among those who had not received the vaccine. Participants who identified as other/non-binary (n=3) were not included in gender analyses due to sample size. Similarly, participants who identified as Asian (n=1), American Indian/Alaska Native (n=3), or multiracial (n=2) were not included in analyses involving race/ethnicity. Bivariate correlations were used to examine associations between COVID-19 vaccination status (yes/no), as well as likelihood of receiving the vaccine among those who had not, and barriers to receiving the vaccine.

## 3 Results

### 3.1 Healthcare use

Most participants (84.3%) had seen a primary care provider and had received an influenza vaccine (65.4%) in the past two years. Almost half (45.4%) reported having a chronic health condition. A majority of participants (67.6%) had already received at least one dose of the COVID-19 vaccine at the time of the survey.

### 3.2 COVID-19 vaccine status by race/ethnicity, sex, age, and education

Overall, female participants were more likely to have received at least one dose of the COVID-19 vaccine (77.6%) compared to male participants (55.7%), *χ* ^2^ (1) = 9.57, *p* <.01. White (73.3%) and Hispanic/Latino (81.8%) participants were also more likely to have received at least one dose of the COVID-19 vaccine than Black/African American participants (54.9%), *χ* ^2^ (2) = 8.86, *p* <.05. Overall, age and education were not associated with vaccination status. See **Table 2**. Among participants who had not received a dose of the COVID-19 vaccine at the time of the survey (n = 57), intention of getting the vaccine was not associated with sex, race/ethnicity, age, or education.

**Table 2:** COVID-19 Vaccine Status x Demographics.

		COVID-19 Vaccine Status	
		% Received at least one dose	% No Dose
**Sex**	Male	55.7	44.3
	Female	77.6	22.4
**Race**	White	73.3	26.7
	Black/African American	54.9	45.1
	Hispanic/Latino	81.8	18.2
**Age**	18-30 years	69.7	30.3
	31-45 years	62.8	37.2
	46-60 years	71.2	28.8
	Over 60 years	74.2	25.8
**Education**	< High School	75.0	25.0
	High School Grad	57.4	42.6
	Some College	74.4	25.6
	Associate Degree	60.0	40.0
	Bachelor’s Degree	84.2	15.8
	Graduate Degree	63.6	36.4

### 3.3 COVID-19 vaccine status by healthcare history

Having a primary care provider was not associated with COVID-19 vaccination status.However, participants who endorsed having seen their primary care provider within the last two years were more likely to have received at least one dose of the vaccine (72.1%) than those who had not (41.7%), *χ* ^2^ (1) = 4.89, *p* <.05. Participants who had received the influenza vaccine in the last two years were more likely to have received at least one dose of the COVID-19 vaccine (80.2%) than those who had not (41.8%), *χ* ^2^ (1) = 25.63, *p* <.001. Those who reported a chronic physical health condition were also more likely to have received at least one dose of the vaccine (77.4%) than those who did not (56.3%), *χ* ^2^ (1) = 8.5.3, *p* <.01.

Endorsement of having previously tested for COVID-19 or knowing someone who tested positive was not associated with vaccination status. Overall ratings of mental or physical health, degree of worry of getting sick with COVID-19, and degree of impact of the pandemic on life and functioning were not associated with vaccination status.

### 3.4 Barriers and attitudes to receiving COVID-19 vaccine

Participants who had not received at least one dose of the COVID-19 vaccine were more likely to report greater concerns about vaccine side effects (*r* = -.42, *p* <.001), vaccine cost (*r* = -.18, *p* <.05), impact on physical health (*r* = -.37, *p* <.001) or medications (*r* = -.39, *p* <.001), and distrust of vaccine manufacturers (*r* = -.48, *p* <.001), distrust of clinicians administering the vaccine (*r* = -.43, *p* <.001), and distrust of federal (*r* = -.38, *p* <.001), state (*r* = -.47, *p* <.001), and local government (*r* = -.45, *p* <.001) as barriers to receiving the vaccine. Among participants who had not yet received the vaccine (*n* = 57), only distrust of federal (*r* = -.40, *p* <.01), state (*r* = -.40, *p* <.001), and local government (*r*= -.34, *p* <.05) was associated with reported likelihood of receiving the vaccine in the future.

## 4 Discussion

The current study found that 67.6% of participants with SMI reported having received at least one dose of a COVID-19 vaccine at the time of survey completion. This vaccination rate is in line with the vaccination rate of the general population of Monroe County during the same time frame. As of June 1, 2021, 55.6% of Monroe County residents had received at least one dose of COVID-19 vaccine. By August 1, 2022, the number had risen to 71.9% (12). This is in line with previous research in Nordic countries showing comparable uptake of COVID-19 vaccination in individuals with and without mental illness (13). This result may be interpreted through both a negative and positive lens. On one hand, given that this population is particularly vulnerable to negative outcomes from COVID-19 infection, it is disappointing that the existing public health initiatives were not able to achieve greater vaccine acceptance in these individuals. On the other hand, the comparable vaccination rate is a testament to the resilience of this population. Having a serious mental illness can pose a significant challenge to accessing health services- symptoms of depression, psychosis, and trauma can interfere with one’s ability to find available care, take public transportation, be in busy public spaces, and navigate a complex mental health system. Despite these challenges, the study population was able to get vaccinated at a comparable rate to the broader local community.

The present study found several demographic differences in the rate of vaccination in the sample population. Female participants were more likely to have received a dose of vaccine (77.6%) compared to male participants (55.7%), *p*<0.01. The current literature is inconsistent with regards to the role of gender in vaccine hesitancy, so it is unclear if these results are representative of a reproducible difference or if this is an intrinsic quality of this cohort. White (73.3%) and Hispanic/Latino (81.8%) participants were more likely to have received at least one dose of the COVID-19 vaccine than Black/African American participants (54.9%). The finding of increased vaccine hesitancy among Black participants with SMI is consistent with previous research showing increased hesitancy among this population in the general population (11). This study did not find significant differences in vaccine acceptance based on age or education level, in contrast to previous research in the general population (11).

This study highlights the importance of active engagement in primary care and preventative healthcare, both of which were associated with vaccine acceptance. Participants who reported having seen their primary care provider within the past two years were more likely to be vaccinated (72.1%) than those who had not (41.7%). Similarly, participants who reported having received an influenza vaccine in the past two years were more likely to be vaccinated (80.2%) than those who had not (41.8%), consistent with previous research (14).

The present study found that individuals with SMI who had not received COVID-19 vaccination were significantly more likely to rate all potential barriers as actual barriers to vaccination. Public health initiatives targeted at educating this group would be well served to better address concerns related to side effects, cost, and distrust of all levels of government. The role of distrust in vaccine hesitancy has been noted in multiple international populations (15). Given that there is no cost for the vaccine itself, future research attempting to better understand financial barriers to receiving care (e.g. transportation or need for childcare) may be fruitful.

There are some limitations of the present study worth noting. First, all data was generated by self-report, thus it was not possible to verify actual vaccination status. The study attempted to ensure survey participant confidentiality and minimize undue influence from investigators by not collecting any identifiable data and by allowing participants to complete the survey in private and without any review or feedback from the researchers. However, by not collecting data about specific symptoms that may affect vaccine hesitancy (e.g. delusional beliefs or negative symptoms), conclusions could not be drawn about the role of active symptoms in vaccine uptake. Interestingly, a recent study in a similar population of individuals with SMI in rural Greece found COVID-19 vaccine refusal was attributable to psychotic symptoms in 30.6% of unvaccinated participants (16). Additionally, interpretation of the results of the present study is limited by evolving vaccine availability over the course of the data collection period, as well as shifting public trends in vaccine acceptance. The present study may underestimate the actual percentage of participants that ultimately received the vaccine. On the other hand, the participants who completed the survey were present on site at a medical facility, selecting for those who may be more active in psychiatric and medical care, thus over-estimating the vaccine acceptance of this population.

Future research on vaccine acceptance among individuals with SMI should focus on engagement strategies for those individuals with the highest degree of vaccine hesitancy, which in this survey were male and Black demographic groups. Further investigation into barriers to healthcare access among marginalized groups may generate actionable interventions to deploy future vaccination campaigns and other public health initiatives. Efforts to enhance active engagement in routine primary care and health maintenance is likely to improve future acceptance of vaccination programs.

## Data Availability

The raw data supporting the conclusions of this article will be made available by the authors, without undue reservation.
